# Development of a Simple Reliable Radiographic Scoring System to Aid the Diagnosis of Pulmonary Tuberculosis

**DOI:** 10.1371/journal.pone.0054235

**Published:** 2013-01-18

**Authors:** Lancelot M. Pinto, Keertan Dheda, Grant Theron, Brian Allwood, Gregory Calligaro, Richard van Zyl-Smit, Jonathan Peter, Kevin Schwartzman, Dick Menzies, Eric Bateman, Madhukar Pai, Rodney Dawson

**Affiliations:** 1 Respiratory Epidemiology and Clinical Research Unit, Montreal Chest Institute, Montreal, Québec, Canada; 2 Department of Epidemiology and Biostatistics, McGill University, Montreal, Québec, Canada; 3 Respiratory Division, Department of Medicine, McGill University, Montreal, Québec, Canada; 4 Division of Pulmonology, Department of Medicine, University of Cape Town Lung Institute, Cape Town, South Africa; 5 University of Cape Town Lung Institute, Cape Town, South Africa; Barcelona University Hospital, Spain

## Abstract

**Rationale:**

Chest radiography is sometimes the only method available for investigating patients with possible pulmonary tuberculosis (PTB) with negative sputum smears. However, interpretation of chest radiographs in this context lacks specificity for PTB, is subjective and is neither standardized nor reproducible. Efforts to improve the interpretation of chest radiography are warranted.

**Objectives:**

To develop a scoring system to aid the diagnosis of PTB, using features recorded with the Chest Radiograph Reading and Recording System (CRRS).

**Methods:**

Chest radiographs of outpatients with possible PTB, recruited over 3 years at clinics in South Africa were read by two independent readers using the CRRS method. Multivariate analysis was used to identify features significantly associated with culture-positive PTB. These were weighted and used to generate a score.

**Results:**

473 patients were included in the analysis. Large upper lobe opacities, cavities, unilateral pleural effusion and adenopathy were significantly associated with PTB, had high inter-reader reliability, and received 2, 2, 1 and 2 points, respectively in the final score. Using a cut-off of 2, scores below this threshold had a high negative predictive value (91.5%, 95%CI 87.1,94.7), but low positive predictive value (49.4%, 95%CI 42.9,55.9). Among the 382 TB suspects with negative sputum smears, 229 patients had scores <2; the score correctly ruled out active PTB in 214 of these patients (NPV 93.4%; 95%CI 89.4,96.3). The score had a suboptimal negative predictive value in HIV-infected patients (NPV 86.4, 95% CI 75,94).

**Conclusions:**

The proposed scoring system is simple, and reliably ruled out active PTB in smear-negative HIV-uninfected patients, thus potentially reducing the need for further tests in high burden settings. Validation studies are now required.

## Introduction

Despite the fact that tuberculosis (TB) is curable, it remains a major problem globally [1]. Central to tuberculosis control programmes is the identification of sputum-positive patients. But smear microscopy has a sensitivity less than 50% among patients with active PTB who are co-infected with HIV [2], and in the HIV era, especially in some countries where more than 70% of patients are HIV-positive, additional methods are required for identifying patients requiring treatment [3]. While much work has been done to optimize sputum microscopy using strategies such as light-emitting diodes [2] and same-day diagnosis [4], in most clinical settings, the chest radiograph remains a central component of the diagnostic work-up, and has not been displaced by recently developed point of care tests, which are limited by both cost and availability [5], [6].

Chest radiography also has significant limitations, particularly when used in the field. In order to be helpful, chest radiographs require both observation and interpretation. Both are subjective, and subject to wide intra- and inter-observer variation [7]. Observation may be improved by a system such as the Chest Radiograph Reading and Reporting System (CRRS) proposed by White *et al* [8]–[10], which ensures systematic recording of features. Interpretation requires knowledge of the appearances and natural history of pulmonary tuberculosis in both HIV infected as well as HIV-negative persons, and agreement between readers often remains poor [7]. Interpretation and utility can potentially be improved by a scoring system that makes the diagnostic decision less arbitrary by prescribing what constitutes a finding, and assigns weights to the features observed on the chest radiograph based on the likelihood of the association of such features with PTB.

Most studies assessing the diagnostic utility of chest radiography have compared the probability of PTB between readers, usually qualified radiologists. In our review of the literature, it was evident that certain features noted on a chest radiograph, such as apical infiltrates and cavities, are known to be highly suggestive of active PTB [11]–[24]. However, we did not find published reports of a simple scoring system that combines the systematic assessment of radiographs for features shown to be relevant to the diagnosis of active pulmonary tuberculosis and that may be suitable for use in the clinic by trained health personnel.

The CRRS was developed to standardize the reading of chest radiographs in epidemiological studies of PTB and lung disease [10], with improved inter- and intra-reader reliability [9], [10]. Although validated for use in epidemiologic studies, its clinical application for the diagnosis of active pulmonary tuberculosis has not been studied. Our study set out to develop a weighted radiographic scoring system to assist interpretation of chest radiographic changes and aid the diagnosis of active pulmonary tuberculosis in the clinical setting.

## Methods

### Study Subjects and Data Collection

The study cohort comprised patients forming part of a large prospective study (TB-NEAT) being conducted at the University of Cape Town (Cape Town, South Africa) to evaluate the performance of new diagnostic tests for tuberculosis [6], [25], [26]. The prevalence of TB in South Africa is estimated to be 795 per 100,000 population and the incidence, 981 per 100,000 population, while the prevalence of HIV infection is 178 per 1000 among adults between the ages of 15 and 49 [27], [28].

Subjects qualified for inclusion in the study if they were ≥18 years and considered by the clinic staff to be patients with possible PTB. To qualify as a patient with possible PTB, an individual had to present to the hospital with at least two of the following symptoms if HIV negative, and one if HIV infected: cough for ≥2 weeks, haemoptysis, fatigue, night sweats, fever for ≥2 weeks, weight loss, loss of appetite, or being bedridden. After giving written informed consent, all patients underwent diagnostic testing, which included two sputum samples evaluated by concentrated smear microscopy, two sputum cultures using the MGIT 960 liquid culture system (BD Diagnostic Systems, Sparks, MD, USA), chest radiography, standardized interferon-gamma release assays, HIV testing, and CD4 T cell count for those who were HIV-infected. Epidemiological data were captured in a questionnaire, which was administered by trained interviewers to all patients.

### CRRS Training

The CRRS training course is held bi-annually at the University of Cape Town Lung Institute (See http://www.lunginstitute.co.za/content/talks.html). The course involves a two and a half-day programme of interactive training using standard chest radiographs. On the first day of the course attendees are instructed on chest anatomy and disease presentation, and a standardized approach to identifying radiological abnormalities is introduced. On the second day attendees read archived radiographs using the structured CRRS form (see online supplement for sample form) and consolidate their understanding about the detail required for standardized reporting. On the third day an examination using 24 standardized radiographs is undertaken and trainees are awarded either “A” or “B grade” accreditation based on their interpretation of an examination set of radiographs.

### Reading of the Chest Radiographs

Chest radiographs were read by two independent readers (BA and GC, specialist physicians undergoing training in pulmonology in the Division of Pulmonology) who had received standard training in the CRRS method, and were blinded to clinical information. Their findings were recorded on a computerized form. The CRRS involves the use of a systematic checklist (Figure S1- online appendix) that details abnormal features visualized on a chest radiograph. These abnormalities are broadly classified into parenchymal, pleural, central and other abnormalities, each of which is further sub-categorized (for example, parenchymal abnormalities are sub-categorized into large opacities, small opacities and cavities). At the conclusion of the examination, the reader is required to provide a subjective assessment of whether the abnormalities recorded are consistent with active TB. In this study, disagreements between readers were resolved through a consensus read by a third senior reader (RVZS, a faculty pulmonologist trained in the use of CRRS). The scoring system was developed using the final single consensus read. Only radiographs performed within 3 months after each subject was enrolled were evaluated.

### Ethics Approval

The study was approved by the University of Cape Town’s Health Sciences Faculty Research Ethics Committee (REC REF 421/2006) and the McGill University Faculty of Medicine ethics committee (Study no. A11-E69-11B).

### Derivation of the Score

The identification of variables to be included in the analysis was guided by a review of the literature [11]–[24]. Features visualized on the chest radiograph suggestive of active PTB that were consistent across the reviewed studies were the presence of lesions in the upper lobes of the lungs, cavities, and unilateral pleural effusions (compared to bilateral effusions) [11]–[24]. These factors were considered *a priori* variables for analysis. Clinical characteristics that were known to influence the interpretation of the radiograph, independent of disease status, were also analyzed. These characteristics included age, sex, smoking status, HIV status and past history of TB.

The outcome of interest was the presence of active PTB, defined as the growth of *M.tuberculosis* on at least one sputum culture. Patients with two negative cultures were classified as having a final culture-negative result. Similarly, a patient with two negative sputum smears was classified as having a smear-negative status.

A univariate analysis was performed to identify significant associations (with a liberal threshold of P<0.2 for statistical significance) between the pre-defined radiographic and clinical features and the outcome of interest. Chi-square tests were used for categorical radiographic variables and *t-*tests were used for continuous variables.

Variables found to be significant in the univariate analysis, or which were identified *a priori* by the literature review were entered into a multivariate logistic regression model. Factors found to be independent predictors of the outcome (P<0.05) were selected for the final model, and a stepwise backward elimination process was employed, using the likelihood ratio test [29], to eliminate variables that did not significantly contribute to the model. We adjusted for HIV status in the final model, as HIV is known to alter the radiographic presentation of active PTB.

We assigned scores to each radiographic feature found to be an independent predictor of outcome in the final model, weighted according to the beta-coefficients from the final multivariate logistic model. Weights were rounded up to the nearest integer.

### Data Analysis

The various major criteria in the CRRS were analyzed for inter-reader reliability between the two initial readers and a kappa statistic for inter-reader agreement was calculated and graded [30], [31]. Based on the weights assigned to the four radiographic features found to be significantly associated with active PTB, we calculated a total score for each patient’s radiograph and analyzed the performance characteristics of the score at various cut-points for the diagnosis of culture-confirmed active PTB. We also analyzed the performance of the score in the subset of patients with smear-negative PTB, and among patients who were HIV-infected. Data were analyzed using STATA version 11.0 (Stata Corp, College Station, Texas, USA).

## Results

### Demographic Characteristics of Subjects

**Figure 1:**
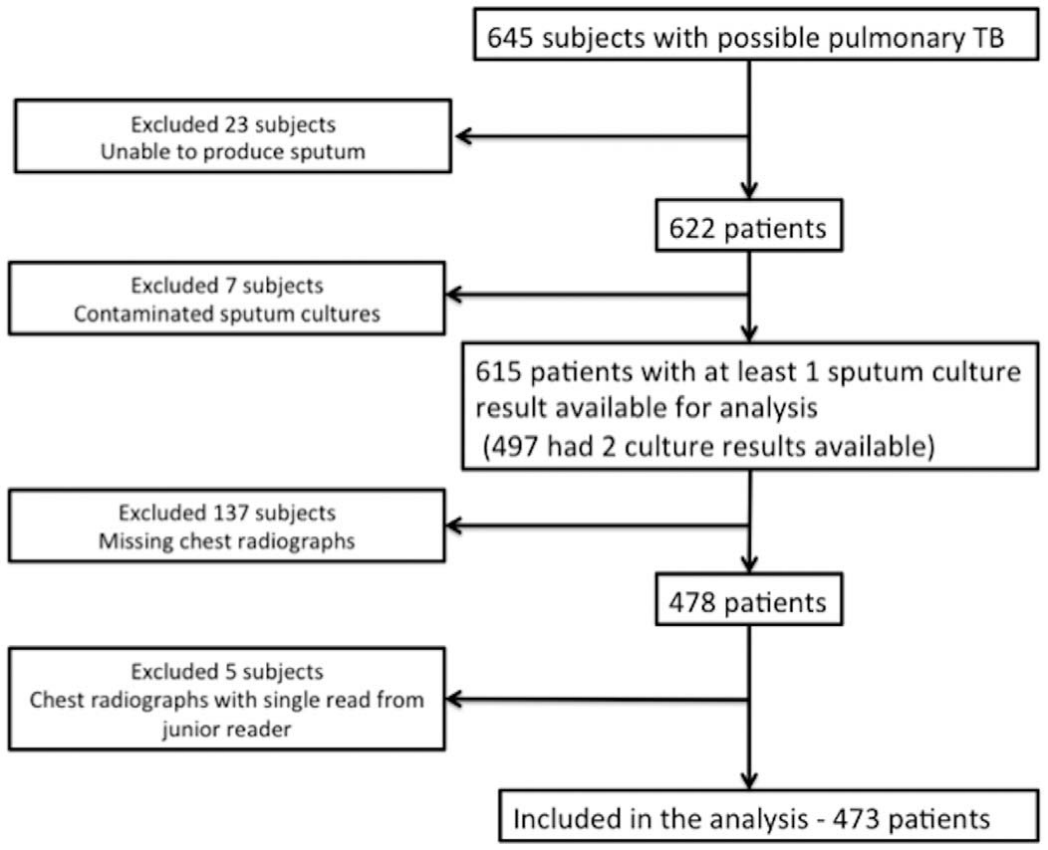
Patient flow diagram for the study.

Of 645 patients recruited into the parent study, 473 patients were included in the final analysis. As outlined in Figure 1, the major reasons for exclusion were inability to produce sputum, contaminated sputum culture, missing chest radiographs, and a chest radiograph read by only one reader. There were no significant differences in the demographic features of the patients who were included and those who were not (Table 1).

**Table 1:** Characteristics of included and excluded patients.

Characteristic		Included patients (n = 473)	Excluded patients (n = 172)	p-value
Age, mean (SD)		39.3(12.1)	39.6(13)	0.79
No. of males,%		329 (69.6)	110 (64)	0.18
Race	black African,%	342 (72.3)	118 (68.6)	0.36
	white/mixed,%	131 (27.7)	54 (31.4)	
HIV status	positive,%	121 (25.6)	52 (30.2)	0.24
	negative,%	285 (60.3)	94 (54.7)	0.2
	unknown/refused,%	67 (14.2)	26 (15.1)	0.77
	median CD4+,IQR	185 (105,349)		
Culture result	positive,%	138 (29.2)	43 (25)	0.29
	negative,%	335 (70.8)	99 (57.6)	
	no results	–	30 (17.4)	
Smear result	at least 1 positive,%	91 (19.2)	35 (20.4)	0.73
	negative,%	382 (80.8)	115 (66.9)	

The mean age of patients was 39.3 (SD 12.1). Sixty-seven patients (14.2%) refused HIV-testing. Of those tested, 121(25.6%) were HIV-infected (median CD4+ cell count = 185/cu.mm among 115 patients with CD4+ counts). Active PTB was confirmed by sputum culture in 138 patients (29.2%) of patients with suspected PTB, and 91(19.2%) of suspect patients were positive on sputum smear microscopy. 47 patients with culture- confirmed PTB were negative on sputum smear microscopy (34% of all patients with PTB).

### Reliability of the CRRS

The inter-reader reliability of the CRRS for various *a priori* major radiographic features of active PTB is summarized in Table 2. The kappa-statistic ranged from moderate (0.56) for small opacities to substantial (0.77) for pleural effusions. The kappa-statistic for the overall judgment on whether the reader considered the features of the chest radiograph to be consistent with active PTB was 0.52 (95% CI 0.42,0.62).

**Table 2:** Inter-reader reliability for the major features reported on the Chest Radiograph Reading and Recording System.

Feature	% agreement	kappa (standard error)
Large opacity (>1 cm)	85.7	0.7 (0.6,0.8)
Small opacity (<1 cm)	92.6	0.56 (0.46,0.66)
Cavity	91.1	0.64 (0.54, 0.74)
Effusion	88.6	0.77 (0.59,0.95)
“Consistent with active TB”*	86.1	0.52 (0.42,0.62)

* This judgment (consistent or not consistent with active TB) was the interpretation of the reader recorded on completion of the CRRS read in the appropriate block on the CRRS form. (see online repository).

### Development of the Score

Results of the univariate analysis of the various chosen radiographic and clinical criteria are summarized in Table 3. All the variables initially selected for inclusion in the multivariable model were found significant, and were therefore retained in the final analysis. The final model was adjusted for age and HIV status, but these were not included in the score, as the aim was to develop a score based only on radiographic features. Based on the beta-coefficients of the variables in the multivariable logistic regression, scores were assigned to the individual radiographic features. The results of the multivariate analysis and scores assigned to the variables in the final model are summarized in Table 3.

**Table 3:** Analysis of radiographic and clinical features in the univariate and multivariable logistic regression model, and weights assigned in the final radiographic score in 473 patients (n = 138 with PTB and 335 without active PTB).

Feature	Patients with PTB with feature n = 138	Patients without PTB with feature n = 335	Crude OR (95%CI),	Adjusted OR (95%CI),	Score assigned
**Large opacity (>1** **cm):**					
UL opacity	108	105	7.9 (5,12.6)	**4.2 (2.1,8.3)**	**2**
ML/LL opacity	116	153	6.3(3.8,10.4)	1.8 (0.9, 3.6)	
**Small opacity (<1** **cm):**					
UL opacity	121	220	3.7 (2.1,6.5)	1.3 (0.6,2.6)	
ML/LL opacity	136	2851	11.9 (2.9,49.8)	2.8 (0.6,13.9)	
**Cavity, any location**	55	23	9 (5.2,15.5)	**4 (2,7.6)**	**2**
**Pleural effusion:**					
Unilateral	35	40	2.5 (1.5,4.2)	**2.1 (1.1,3.9)**	**1**
Bilateral	1	4	0.6 (0.7,5.5)		
**Apical cap**	53	69	2.4(1.6,3.7)	0.8 (0.4,1.5)	
**Adenopathy, any location**	23	21	3 (1.6,5.6)	**3.8 (1.7,8.2)**	**2**
**Tracheal deviation/Mediastinal shift/Hilar elevation**	31	27	3.3 (1.9,5.8)	1.2(0.6,2.4)	
**HIV infection**	43	78	1.5 (1, 2.3)	1.3 (0.7,2.2)*	
**Sex - males**	100	229	1.2 (0.8,1.9)		
**Smoker current**	79	196	0.9 (0.6,1.4)		
**Smoker past**	18	35	1.3 (0.7,2.4)		
**Smoker ever**	133	318	1 (0.6,1.5)		
**Age mean, SD**	36.84 (11.55)	40.26 (12.15)	0.98 (0.96,0.99)	0.96 (0.94,0.98)*	

* adjusted for, but not assigned weights in the final model. The final model included UL large opacity, cavity (any location), unilateral pleural effusion and adenopathy (any location).

### Performance of the Score

The score thus developed was tested at different cut-offs, the results of which are shown in Table 4. At a cut-off of ≥2, the score had a high negative predictive value (91.5%, 95%CI 87.1–94.7), and misclassified 20 of 138 patients with active PTB. The score improved the specificity of the test at this cut-off (63.9%, 95% CI 58.5–69) as compared to the specificity of the subjective assessment of the probability of PTB by the readers (27.5%, 95% CI 22.6,32.8) with a loss in sensitivity that was not statistically significant (85.5, 95% CI 78.5,90.9 v/s 93.4, 95% CI 87.9,97) (Table 4). The positive likelihood ratio (LR+) for the test at this cut-off was 2.37 and the negative likelihood ratio (LR-) was 0.23. The gain in specificity at higher cut-offs for the score was accompanied by appreciable losses in sensitivity.

**Table 4:** Performance characteristics of the score at different cut-offs in 473 patients (n = 138 with PTB and 335 without PTB).

Cut-off	Sensitivity	Specificity	PPV	NPV	Area under the ROC curve**
≥1	89.1(82.7,93.8)	58.2(52.7,63.5)	46.8(40.6,53)	92.9(88.5,95.9)	0.74(0.7,0.77)
**≥2**	**85.5(78.5,90.9)**	**63.9(58.5,69)**	**49.4(42.9,55.9)**	**91.5(87.1,94.7)**	**0.75(0.71,0.79)**
≥3	57.2(48.5,65.6)	86.9(82.8,90.3)	64.2(55.1,72.7)	83.1(78.8,86.9)	0.72(0.68,0.77)
≥4	13.8(8.5,20.7)	98.5(96.6,99.5)	79.2(57.8,92.9)	73.5(69.2,77.5)	0.56(0.53,0.59)
**“Consistent with active TB” reported by readers** *	93.4(87.9,97)	27.5(22.6,32.8)	36.1(31.1,41.3)	90.5(82.8,95.6)	0.6(0.57,0.64)

Calculated Score = (UL large opacity*2)+(cavity, any location*2)+(unilateral pleural effusion*1)+(adenopathy, any location*2).

* This judgment (consistent or not consistent with active TB) was the interpretation of the reader recorded on completion of the CRRS read. in the appropriate block on the CRRS form. (see online repository).

** The various AUROCs were derived from a dichotomous test at each cut-point.

In sputum-smear negative patients, at the same cut-off, the test had a good rule-out value (NPV 93.4%, 95% CI 89.4,96.3). 214 of 382 smear-negative patients were correctly classified by the score as not having active disease, and 15 smear-negative patients with the disease were incorrectly classified by the score. The performance of the score in smear-negative PTB patients is shown in Table 5. The score had a better negative predictive value for HIV-uninfected individuals (92.1, 95% CI 86.3, 96) than in HIV-infected individuals (86.4, 95% CI 75,94) (Table 6), although the difference was not statistically significant (p = 0.21).

**Table 5:** Performance of scoring system among smear-negative patients in comparison to checklist-based diagnosis (n = 47 with PTB and 335 without PTB).

Performance characteristics	Sensitivity	Specificity**	PPV	NPV	Area under ROC curve
Checklist-based diagnosis amongsmear negative patients*	83.3 (69.8,92.5)	27.7 (22.8,33)	15.1 (11,20)	91.5 (83.9,96.3)	0.55 (0.5,0.61)
Score at cut-off ≥2 amongsmear-negative patients	69.4 (54.6,81.7)	64.3 (58.9,69.4)	22.2 (15.9,29.6)	93.4 (89.4,96.3)	0.67 (0.6,0.74)

* This judgment (consistent or not consistent with active TB) was the interpretation of the reader recorded on completion of the CRRS read. in the appropriate block on the CRRS form. (see online repository).

** p-value for the difference in specificity <0.001.

**Table 6:** Performance of scoring system among HIV-infected and uninfected patients (121 HIV-infected patients, 285 HIV-uninfected patients).

Performance characteristics	Sensitivity	Specificity*	PPV	NPV	Area under ROC curve
Score at cut-off ≥2 amongHIV-positive patients	81.4 (66.6,91.6)	65.4 (53.8,75.8)	56.5 (43.3,69)	86.4 (75,94)	0.73 (0.65,0.81)
Score at cut-off ≥2 amongHIV-negative patients	86.2 (76.7,92.9)	62.4 (55.4,69)	47.3 (38.9,55.7)	92.1 (86.3,96)	0.74 (0.69,0.79)

* p-value for the difference in specificity = 0.21.

## Discussion

We have developed a scoring system for chest radiographs for use in patients being investigated for active PTB, that performs satisfactorily as a rule-out test in both smear-positive and smear-negative patients. Although the score was developed using radiographic features reported by trained clinicians employing a validated method for reporting on chest radiographs (the CRRS system), the score is based on 4 easily recognized features – the presence of upper lobe opacities, cavities, a unilateral pleural effusion, and hilar or mediastinal lymphadenopathy, features that have been consistently reported in the literature to be associated with active PTB [11]–[24]. While not useful for confirming the presence of active PTB, the scoring system may at least be suitable for ruling out active disease and reduce the use of more expensive diagnostic tests. Since it is not intended to replace, but to supplement the use of sputum smear examination, it is reassuring that it performs satisfactory in smear-negative and in patients infected with HIV, in whom active PTB is often associated with a negative smears. This potential application needs to be examined in a prospective study performed in the field setting.

The proposed score has limitations. Firstly, a rule-out test is less useful to clinicians than one that confirms active PTB. Its primary purpose is to reduce the need for further confirmatory tests and/or referral for further examination, which can be useful in resource poor settings, particularly where access to clinics and investigations is limited. In such circumstances, saving patients the need to visit distant facilities may be valuable.

The current score differs from other diagnostic scoring systems developed to assist in the diagnosis of active PTB, in that it does not include clinical data. Most existing methods include the presence or absence of symptoms and clinical features of PTB, with a radiographic score [11]–[24]. A recent systematic review of such scoring systems showed that eleven of the thirteen studies found were used for making decisions concerning hospital respiratory isolation, and only one study assessed a scoring system for out-patients. All of the scores were dependent on clinical features in patients, and were found to be very sensitive, but poorly specific [32]. Although their performance in diagnosing active disease is useful, a limitation is that the clinicians collecting the clinical data might not feel confident in evaluating the chest radiograph. With our method, a health worker trained in the CRRS methodology may be better able to provide a report to the attending clinician that will influence the clinical decision in sputum negative patients, on whether to further evaluate a patient with more diagnostic tests, or to follow-up the patient clinically. It is recognized however that in our study patients were selected on the basis of one or more symptoms or features consistent with the diagnosis of active PTB. Thus the pretest probability of a positive diagnosis was increased. However, this reflects the setting in which the test might be used in clinical practice.

A further limitation of the method, is that its negative predictive value in HIV-infected patients (86.4, 95% CI 75–94), may not be sufficiently high in this highly vulnerable group, in whom delayed diagnosis is associated with rapid progression of disease and death [33]. This imprecision of the negative predictive value may be a reflection of the small sample size. It is also possible that the insufficiently high NPV is a reflection of the reported fact that a significant proportion (over 10%) of HIV-infected individuals with clinical symptoms consistent with PTB can have normal chest radiographs [34]. Further studies will have to be conducted to assess the utility of the scoring system in this category of patients.

The finding of high inter-reader reliability for the major features among readers trained to report radiographs using the CRRS is consistent with earlier reports [8]–[10]. The high reliability is useful, given that standardizing the reading of chest radiographs for PTB and increasing the reproducibility has always been an impediment to the accuracy of the test. Our study supports the role of the CRRS system, which was designed as a tool for epidemiological surveys, as an aid to clinical decision-making. The work reported here was based on reports generated by trained readers with considerable clinical experience in chest radiology. They had received the 2 and a half-day intensive training required for CRRS accreditation. Whether this simplified clinical score will perform as well when radiographs are read by non course- trained observers needs to be tested in prospective studies. Even a simplified score will require some degree of training and standardization before it can be widely implemented.

Our study has several limitations. Due to logistical difficulties, 8 patients (1.7%) had chest radiographs that had discordant readings that were not resolved by a third reader, and we had to exclude 5 of these patients who did not have radiographs reported by the third reader for analysis. Secondly, several patients had to be excluded from the analysis, primarily due to the absence of chest radiographs, and this might have introduced bias. However, the comparisons of the demographic features of the patients included and excluded suggest that this was unlikely. Thirdly, we tested the score in the same population from which the score was derived, and this could lead to an overestimation of its performance. However, the consistency of the features used in the score with what is described in the literature suggests that these features are likely to be reproducible. Despite the high negative predictive value of the score, misclassification (false negatives) occurred in 20 patients with active disease. This highlights the principle that all diagnostic tests including the chest radiograph must be interpreted within the clinical context of the case, and appropriate advice given and follow-up arranged for patients who have progressive or ongoing symptoms. Finally, we acknowledge the fact that performance characteristics of diagnostic tests are influenced by prevalence of disease, and the external validity of the scoring system can only be established after using it in settings with different burdens of TB and HIV, and the consequent varying radiographic presentations of the disease.

In conclusion, we have developed a scoring system that attempts to optimize the observation and interpretation of chest radiographs for the diagnosis of PTB. The system uses the CRRS, a tool with high inter-reader reproducibility, for observing and documenting the abnormalities visualized on the chest radiograph. For interpretation of these abnormalities, we have developed a simplified score based on assigned weights to four easily recognized features on the chest radiograph. The system thus developed has a high negative predictive value, making it a useful tool to rule out active PTB in persons with negative sputum smears, especially in patients who are not infected with HIV. Further validation studies are now necessary to confirm our findings.

## Supporting Information

Figure S1 **Chest Radiograph Reading and Recording (CRRS) form.**
(JPG)
