# Supplementary figures and images for: Development of a Simple Reliable Radiographic Scoring System to Aid the Diagnosis of Pulmonary Tuberculosis
Source: PLoS One. 2013 Jan 18;8(1):e54235. doi: 10.1371/journal.pone.0054235 (PMC3548832; doi:10.1371/journal.pone.0054235)

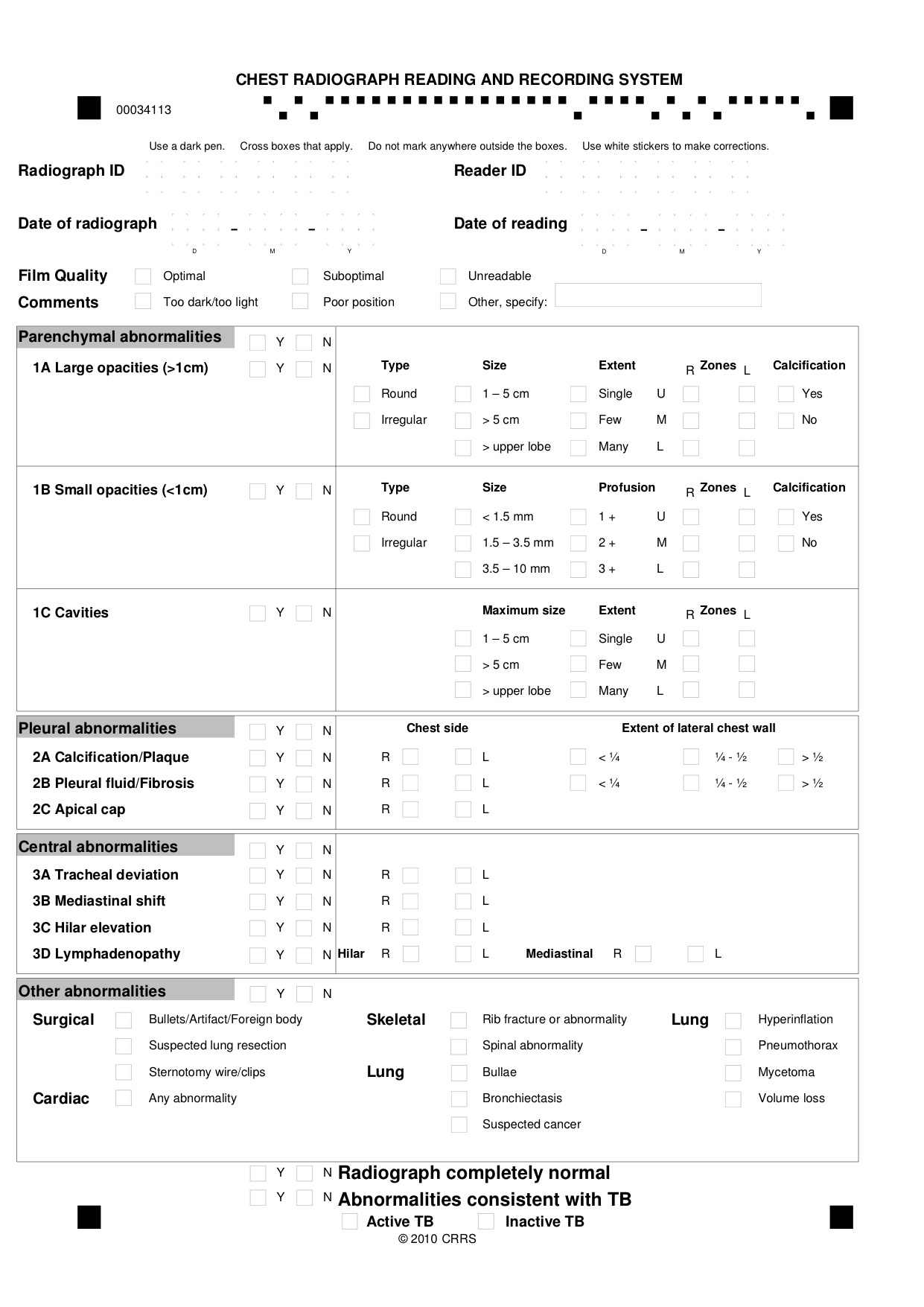

Supplement: Figure S1 — Chest Radiograph Reading and Recording (CRRS) form. (JPG) [file pone.0054235.s001.jpg]
